# The Use of Cocaine as an Anæsthetic

**Published:** 1891-07

**Authors:** 


ARTICLE VII.

### THE USE OF COCAINE AS AN ANÆSTHETIC.

At a meeting of the Academie de Medecine, on May 12th, the report of the commission appointed to consider M. Hallopeau's communication on cocainism (see supplement, December 13th 1890, p. 84), was presented by MM. Panas and Magitot (*Bull. de l' Acad. de Med.*, 3me Serie, t. xxv. No. 19). After a review of the literature of the subject—confined almost exclusively to French sources—they cite the experience of M. Paul Reclus, who has performed more than 1,200 operations (removal of tumors, hydrocele, excision and erasion of joints, laparotomy, gastrostomy, strangulated hernia, etc.) without accident. M. Reclus finds that for mucus membranes the application of cocaine to the surface is sufficient, but if more profound anæsthesia is required it should be injected, not hypodermically, but intradermically. Care must be taken not to inject any of the drug into a vein; the injection should be made slowly, the quantity never exceeding 10 centigrammes, this amount being used only for extensive operations, and being administered in a series of injections at different points so as to embrace a wide surface. MM. Panas and Magitot, following in great part the teaching of M. Reclus, sum up their conclusions as follows: (1) Cocaine is an excellent local anæsthetic, which should on no account be banished from surgical practice. (2) It should, however, be used with the following precautions: (*a*) The dose should be proportional to the extent of surface it is desired to render insensitive, but should in no case exceed from 8 to 10 centigrammes; (*b*) cocaine should never be employed in persons suffering from cardiac complaints, chronic affections of the respiratory passages, or well-marked neurotic diathesis; (*c*) it should be injected *into* and not under the mucus membrane and skin; a 2 per cent. solution is the best; it should always be prepared fresh and injected with a sterilized syringe; (*d*) the patient should always be in the recumbent position when the injection is given, even if the head has to be raised afterwards; (*e*) the drug should be of the most absolute purity, and in particular free from any admixture of toxic alkaloids; (*f*) it should be given in divided doses, an interval of some minutes being allowed to elapse after the first injection of the first portion; this will give an opportunity of observing whether any untoward effect is likely to occur. (3.) Used in this way cocaine has obvious advantages over chloroform, ether, etc., one of the chief being the possibility of operating without assistance. (4.) The anæsthetic effect always lasts long enough to allow of the performance of "all the operations of ordinary surgery." In the discussion which followed the reading of the report M. Constantin Paul spoke of the internal administration of cocaine, pointing out that doses of 15, 20, and even 30 centigrammes could be taken in this way without danger. He said the intradermic injection was very painful. M. Laborde, while admitting that much larger doses could be taken by the mouth than could be safely given hypodermically, and that remarkable analgesic effects could thus be produced in cases of gastralgia, etc., and that he had seen serious accidents from the internal administration of large doses. The conclusions embodied in the report of MM. Panas and Magitot were adopted by the Academy—*Brit. Medical Journal.*

